# Dual-trajectory Approach for Simultaneous Cyst Fenestration and Endoscopic Third Ventriculostomy for Treatment of a Complex Third Ventricular Arachnoid Cyst

**DOI:** 10.7759/cureus.253

**Published:** 2015-03-05

**Authors:** Allen L Ho, Arjun V Pendharkar, Eric S Sussman, Vinod K Ravikumar, Gordon H Li

**Affiliations:** 1 Department of Neurosurgery, Stanford University School of Medicine; 2 Department of Neurosurgery, Stanford University Medical Center; 3 Department of Neurosurgery, Stanford School of Medicine/Stanford University Medical Center

**Keywords:** arachnoid cyst, endoscopic third ventriculostomy, burrhole, hydrocephalus, image guidance

## Abstract

Objectives: We present a case of a multiloculated third ventricular arachnoid cyst to describe a novel technique for definitive management of these lesions via direct endoscopic fenestration and CSF diversion utilizing separate trajectories that offers superior visualization and avoids forniceal injury.

Methods and Results: We present a case of a 33-year-old woman with progressive headache and worsened vision, a known history of a multiloculated third-ventricular arachnoid cyst, and imaging findings consistent with cyst expansion and worsened obstructive hydrocephalus. We then describe the dual-trajectory approach for simultaneous cyst fenestration and endoscopic third ventriculostomy that ultimately resulted in successful treatment of her cyst and hydrocephalus.

Conclusions: Dual-trajectory endoscopic approach utilizing double burr holes should be considered when addressing lesions of the third ventricle causing obstructive hydrocephalus.

## Introduction

Estimates of the prevalence of arachnoid cysts in adults vary anywhere from 0.2 to 1.7% [1]. Cysts located in the third ventricle can cause obstructive hydrocephalus as a result of compression or direct occlusion of the cerebral aqueduct or foramen of Monro. They have also been shown to cause endocrine dysfunction and/or visual impairment via mass effect on the optic tracts or pituitary axis. CSF diversion via implanted shunting systems is effective at addressing the hydrocephalus associated with these cysts; however, these systems are associated with both mechanical and infectious complications, often necessitating multiple revisions [2-8]. While transcallosal craniotomy can achieve definitive treatment of the cyst via fenestration and/or resection and can aid in avoiding shunt dependence, the morbidity of an open approach is not insignificant and includes damage to crucial vascular structures, disconnection syndromes from splitting the corpus callosum, and damage to the fornices and subcortical nuclei [9]. Several studies have now described successful management of arachnoid cysts with endoscopic approaches [10-13]. However, in many of these cases, especially with multi-loculated lesions, the need for revision fenestrations or permanent CSF diversion was still necessary [9, 14-15]. Because of this, emphasis has been placed on considering a combination of procedures to minimize recurrence and achieve successful treatment of these complex lesions [13, 16-18]. We describe a novel technique for definitive management of these lesions via direct endoscopic fenestration and CSF diversion utilizing separate trajectories that avoids forniceal injury.

### Case presentation

**Figure 1:**
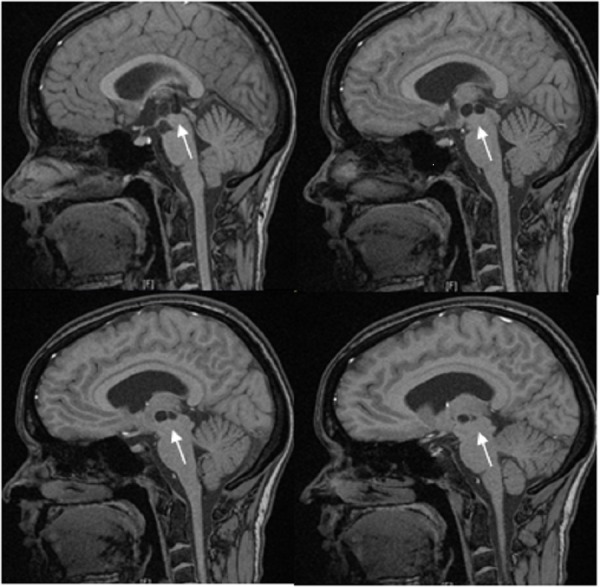
Patient imaging five years prior to initial presentation T1 weighted, non-contrast, sagittal brain MRI demonstrating a multi-loculated third ventricular cystic structure (arrows).

**Figure 2:**
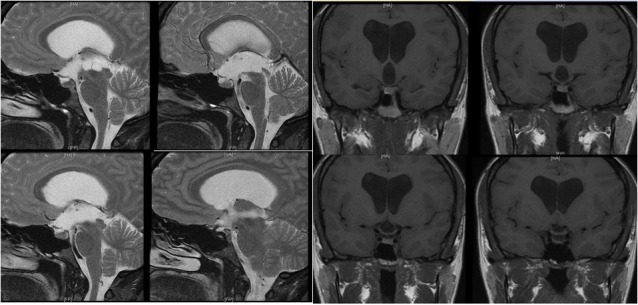
Patient imaging at presentation (Left) T2 weighted sagittal brain MRI demonstrating an enlarged multi-loculated cystic structure located in the third ventricle with worsened ventriculomegaly. (Right) T1 weighted non-contrast coronal brain MRI demonstrating an enlarged multi-loculated cystic structure located in the third ventricle with worsened ventriculomegaly.

The patient is a 33-year-old woman who presented with six months of progressive headaches (worse in the morning) and blurred vision. She had a history of galactorrhea and a diagnosis of hydrocephalus and a third-ventricular cyst based off of a MRI scan done five years prior, but ultimately did not seek any follow-up care (Figure 1). Her neurologic exam was unremarkable except for papilledema. MRI demonstrated worsened ventriculomegaly and an enlarged complex cystic structure within the third ventricle consistent with an arachnoid cyst (Figure 2). The patient underwent a dual-trajectory, double burr hole approach for simultaneous cyst biopsy and fenestration and endoscopic third ventriculostomy (ETV). Final pathology was consistent with an arachnoid cyst. Postoperatively, the patient did well. All her symptoms resolved, and she remained symptom-free at her six-month follow-up.

## Technical report

Several neurosurgical treatment options exist for hydrocephalus secondary to third-ventricular arachnoid cysts, including CSF diversion via a ventriculoperitoneal shunt, open surgical resection of the cyst, or a less invasive endoscopic approach. We elected to utilize a double burr hole endoscopic approach combined with image guidance in order to address the cyst via direct endoscopic fenestration and create a channel for CSF diversion via an endoscopic third ventriculostomy (ETV).

Informed patient consent was obtained prior to treatment.

**Figure 3:**
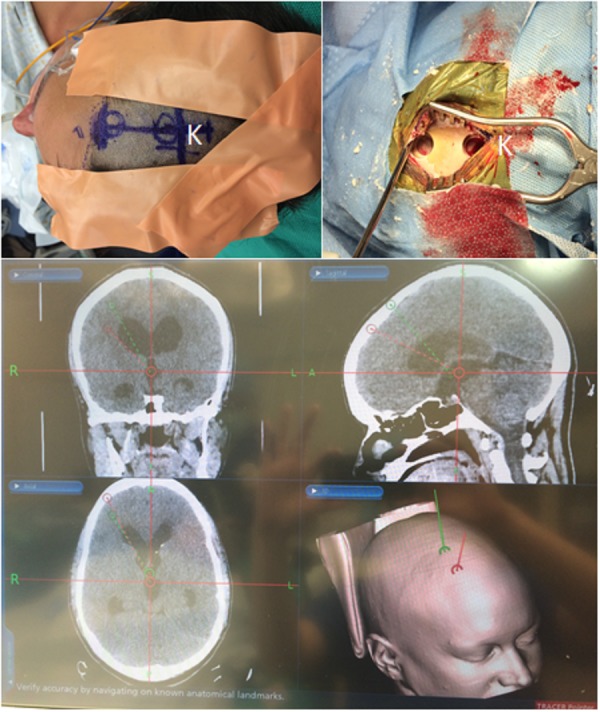
Dual-trajectory operative approach (Top) Double burr hole placement at Kocher’s point (K) and extended anteriorly in the mid-pupillary line to just behind the hairline to facilitate the dual-trajectory operative approach. (Bottom) Planned dual-trajectories for ETV (green) and endoscopic third ventricular cyst fenestration (red) utilizing the StealthStation® AxiEM^TM^ frameless image-guided surgical navigation system.

After endotracheal intubation, the patient was placed supine on the operating table under general anesthesia. The head was secured in a gel donut in the supine position. The StealthStation® AxiEM^TM^ frameless image guidance system (Medtronic, Minneapolis, MN, USA) was initiated with CT imaging and facial registration. A standard trajectory was planned with the entry point at Kocher’s point for the ETV. However, the approach to the cyst necessitated a more anteriorly placed entry point so that the endoscope could be directed posteriorly to the cyst without stretching the fornices. The second entry point was determined utilizing the trajectory view while planning to optimize direct visualization of the cyst. An incision was made at the mid-pupillary line on the right side over Kocher’s point that was extended anteriorly to the end of the hairline. Burr holes were made at both Kocher’s point and anteriorly at the hairline (Figure 3).

**Figure 4:**
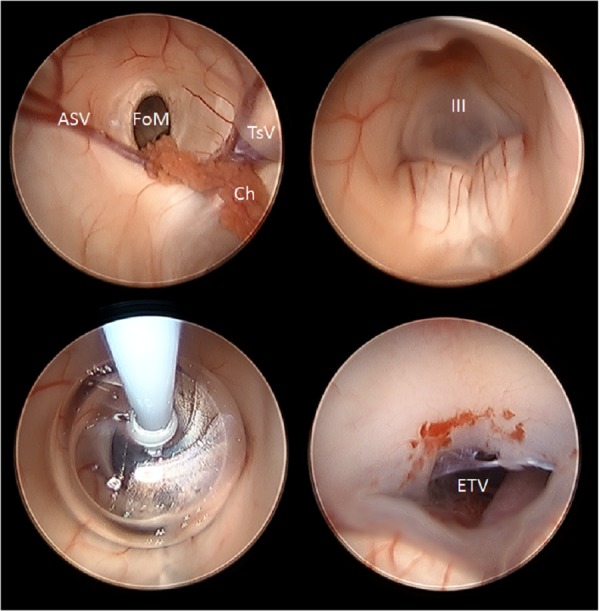
Endoscopic third ventriculostomy (ETV) Intraoperative neuroendoscopy image captures demonstrating visualization of the foramen of Monro (top left, FoM) with anterior septal vein (ASV), choroid (Ch) and thalamostriate vein (TsV) landmarks, visualization of the floor of the third ventricle (III) through the foramen Monro (top right), creation of third ventriculostomy via dilation of the NeuroBalloon^TM^ (bottom left), and completed ETV (bottom right, ETV).

Out of concern that the biopsy/fenestration of the cyst could lead to hemorrhage that would interfere with ETV, we chose to perform the ETV first since CSF diversion would most directly address her symptomatic hydrocephalus. AxiEM^TM^ guidance was used to place a 19 French peel away sheath catheter into the ventricle through Kocher’s point on first pass. A MINOP® endoscope (Aesculap Inc., Center Valley, PA, USA) was placed through the peel away sheath, and the foramen of Monro was identified with choroid plexus and thalamostriate vein landmarks. The floor of the third ventricle was then identified through the foramen of Monro. A bugbee wire was utilized to fenestrate the floor of the third ventricle anterior to the mammillary bodies, and an Integra NeuroBalloon^TM^ catheter (Integra LifeSciences Corp., Plainsboro, NJ, USA) was placed in the opening and inflated to create the ventriculostomy as previously described (Figure 4) [19].

**Figure 5:**
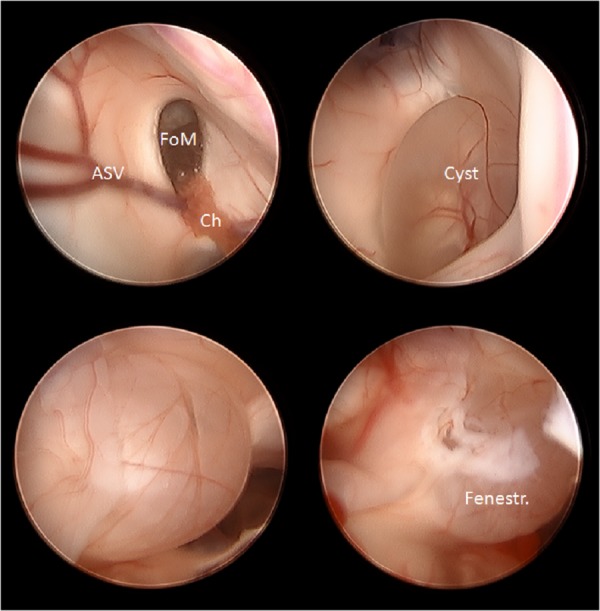
Endoscopic arachnoid cyst fenestration Intraoperative neuroendoscopy image captures demonstrating visualization of the foramen of Monro (FoM) with choroid (Ch) and anterior septal vein (ASV) landmarks (top left), direct visualization of the posterior third ventricle and overlying arachnoid cyst (top right and bottom left, Cyst), and fenestration of arachnoid cyst (bottom right, Fenestr.).

Next, we addressed the cyst via the anterior burr hole. A 19 French peel away catheter was passed with AxiEM^TM^ guidance from an anterior to posterior trajectory. The endoscope was placed in the Kocher’s point entry in the lateral horn to visualize the new catheter as it was entering the lateral ventricle. The endoscope was then placed through the anterior burr hole and the foramen of Monro was again visualized, but this time from a more anterior approach. After entering the foramen, we could visualize a clear, benign-appearing arachnoid cyst in the posterior third ventricle. A bugbee wire was utilized to fenestrate the cyst multiple times, and specimens were sent for permanent pathology (Figure 5). At the conclusion of the procedure, hemostasis was achieved with irrigation, and the burr holes were filled with gel foam and covered with Snythes burr hole covers. The incision was closed in a standard fashion. Postoperatively, the patient remained neurologically intact and memory was preserved.

## Discussion

We report on a dual-trajectory endoscopic technique for treatment of a complex third-ventricular arachnoid cyst via direct cyst fenestration and third ventriculostomy utilizing separate trajectories that avoids forniceal injury. Endoscopic arachnoid cyst fenestration is a well-validated technique for successful treatment of symptoms of hydrocephalus resulting from these lesions [10-13]. However, more complex, multi-loculated lesions often require additional revision fenestrations or permanent CSF diversion [9, 14-15]. Thus, combination procedures with either permanent CSF shunt placement or ETV have been advocated for treatment of more complex lesions [13, 16-18].

Aside from hemorrhage, neural injuries are the second most common complication of endoscopic third ventriculostomies (1.44%). Forniceal injury is one of the most well described neurologic injuries resulting from ETV, though the reported incidence in the literature is quite low (0.04%) [20]. It is caused by direct shearing forces on the fornices during passage of the endoscope through the foramen of Monro and results in memory disorders seen after ETV (0.17%) [21-23]. Utilization of a second burr hole and endoscope trajectory in the case of a posteriorly located arachnoid cyst of the third ventricle allows us to achieve the dual goals of cyst fenestration and CSF diversion with minimal risk of forniceal injury.

## Conclusions

Dual-trajectory endoscopic approach utilizing double burr holes should be considered when addressing lesions of the third ventricle causing obstructive hydrocephalus. This approach optimizes visualization and decreases shearing injury to the fornices and other surrounding structures to achieve the goals of both addressing the lesion and creating permanent CSF diversion via ETV.
